# Metabolic Factors Associated with Risk of Renal Cell Carcinoma

**DOI:** 10.1371/journal.pone.0057475

**Published:** 2013-02-28

**Authors:** Christel Häggström, Kilian Rapp, Tanja Stocks, Jonas Manjer, Tone Bjørge, Hanno Ulmer, Anders Engeland, Martin Almqvist, Hans Concin, Randi Selmer, Börje Ljungberg, Steinar Tretli, Gabriele Nagel, Göran Hallmans, Håkan Jonsson, Pär Stattin

**Affiliations:** 1 Department of Surgical and Perioperative sciences, Urology and Andrology, Umeå University, Umeå, Sweden; 2 Institute of Epidemiology and Medical Biometry, Ulm University, Ulm, Germany; 3 Institute of Preventive Medicine, Copenhagen University Hospital, Copenhagen, Denmark; 4 Department of Surgery, Skåne University Hospital, Lund University, Malmö, Sweden; 5 Department of Public Health and Primary Health Care, University of Bergen, Bergen, Norway; 6 Norwegian Institute of Public Health, Oslo/Bergen, Norway; 7 Department of Medical Statistics, Informatics and Health Economics, Innsbruck Medical University, Innsbruck, Austria; 8 Agency for Preventive and Social Medicine, Bregenz, Austria; 9 Institute of Population-based Cancer Research, The Cancer Registry of Norway, Oslo, Norway; 10 Department of Public Health and Clinical Medicine, Nutritional Research, Umeå University, Umeå, Sweden; 11 Department of Radiation Sciences, Oncology, Umeå University, Umeå, Sweden; 12 Department of Surgery, Urology Service, Memorial Sloan-Kettering Cancer Center, New York, New York, United States of America; Dartmouth, United States of America

## Abstract

Previous studies have shown that obesity and hypertension are associated with increased risk of renal cell carcinoma (RCC), but less is known about the association to other metabolic factors. In the Metabolic Syndrome and Cancer project (Me-Can) data on body mass index (BMI, kg/m2), blood pressure, and circulating levels of glucose, cholesterol, and triglycerides were collected from 560,388 men and women in cohorts from Norway, Austria, and Sweden. By use of Cox proportional hazard models, hazard ratios (HR) were calculated for separate and composite metabolic exposures. During a median follow-up of 10 years, 592 men and 263 women were diagnosed with RCC. Among men, we found an increased risk of RCC for BMI, highest vs. lowest quintile, (HR = 1.51, 95% CI 1.13–2.03), systolic blood pressure, (HR = 3.40, 95% CI 1.91–6.06), diastolic blood pressure, (HR = 3.33, 95% CI 1.85–5.99), glucose, (HR = 3.75, 95% CI 1.46–9.68), triglycerides, (HR = 1.79, 95% CI 1.00–3.21) and a composite score of these metabolic factors, (HR = 2.68, 95% CI 1.75–4.11). Among women we found an increased risk of RCC for BMI, highest vs. lowest quintile, (HR = 2.21, 95% CI 1.32–3.70) and the composite score, (HR = 2.29, 95% CI 1.12–4.68). High levels of the composite score were also associated with risk of death from RCC among both men and women. No multiplicative statistical or biological interactions between metabolic factors on risk of RCC were found. High levels of BMI, blood pressure, glucose and triglycerides among men and high BMI among women were associated with increased risk of RCC.

## Introduction

The highest incidence of renal cell carcinoma (RCC) is found in North America and Europe and the incidence has been increasing world-wide until recently [1], [2]. The increase can be partly explained by improved detection by use of ultra sound and magnetic resonance imaging [3], but it may also be due to an increasing prevalence of risk factors [1].

Established life-style related risk factors for RCC are obesity, hypertension, and smoking [1], [2], and these risk factors have been estimated to account for up to 50% of the cases [4]. Previous studies have reported that diabetes type 2 among women [5] and high BMI and blood pressure among men [6] are independent risk factors for RCC, however, those studies had no data of blood lipids, which may be a mediator of these associations. Another study reported that high levels of triglycerides were associated with risk of RCC [7], and found that the association was stronger among obese subjects, however, no data for smoking or hypertension was included in that study.

Thus, less is known about lipids [7], [8] and glucose [7], [9] and it is also unclear if any of the metabolic factors independently increase the risk, or if they are part of the same pathway, or interact on risk of RCC. Most studies for metabolic factors and risk of RCC have used dichotomized levels of exposure, however, it remains to be shown if there is a threshold with a distinct risk increase, or if the association between increasing levels of metabolic factors and risk is linear.

The aim of this study was to investigate the associations between metabolic factors, separately and jointly, and the risk of RCC and death from RCC taking random measurement error into account.

## Materials and Methods

### Study Population

This study was conducted within the Metabolic syndrome and Cancer project (Me-Can), which has previously been described in detail [10]. In brief, the Me-Can project consists of pooled cohorts in Norway, Sweden and Austria, and in the current study we used 560,388 subjects with complete data on body mass index (BMI, weight/height^2^; kg/m^2^), systolic and diastolic blood pressure, and circulating levels of glucose, cholesterol, and triglycerides from one or several health examination(s). Measurements from the baseline health examination were used in the main analysis. The study was approved by The Research Review Board of Umeå, Sweden, the Regional Committee for Medical and Health Research Ethics, Southeast Norway and the Ethikommission of the Land Vorarlberg, Austria. Participants from Sweden and Austria provided written informed consent to participate in this study. In Norway, the participants were invited to come to the health survey and a questionnaire was sent together with the invitation. An attendance to the health examination where the participants delivered their filled in questionnaire, has been accepted by the Data Inspectorate as an informed consent, but not a written consent. Written consent was obtained from 1994.

### End-points

Cancer diagnoses were identified through linkages with the National cancer Registry in Norway and Sweden and to the Vorarlberg state cancer registry in Austria. The International Classification of Diseases, seventh revision (ICD-7) codes 180.0 and 180.9 was used for identification of RCC cases. Causes of death were coded according to Eurostat European shortlist for causes of death [11] and were obtained by linkage to National Cause of Death Registry in each country. In Norway and Sweden, data were also linked to the Registry of Total Population and Population Changes for assessment of vital status (data not available in Austria). In order to reduce the probability of reverse causation, follow up started one year after baseline health examination.

### Statistical Methods

Risk was analysed with Cox proportional hazards regression with age as time scale. Subjects were followed until the date of event, i.e. RCC diagnosis or RCC death, or until censoring at the date of other cancer diagnoses (for incident analysis only), death, emigration, or end of follow-up up (for analysis of RCC diagnosis: December 31, 2003 in Austria, 2005 in Norway, and 2006 in Sweden, for analysis of RCC death: December 31, 2003 in Austria, and 2004 in Norway and Sweden), whichever occurred first. Hazard ratios (HRs) were calculated for quintiles of exposure and for exposures transformed to standard scores (z-scores).

HRs were calculated for exposures in quintiles with the lowest quintile as reference. The median value of each quintile was used to test for linear trend across quintiles. Quintile analysis were stratified for cohort and adjusted for age at measurement, categories of birth year (before 1923, 1923–1930, 1931–1938, 1939–1946, 1947–1954, 1955 and later) and were adjusted for smoking status (current/never/former smoker) and quintiles of BMI (except for BMI and the composite score).

To convert the exposures to the same scale, we transformed the original values to standardized variables (z-scores) with zero as mean and one as standard deviation. Glucose, triglycerides and cholesterol were divided into quintiles and transformed into z-scores separately for cohort, sex, and fasting time (<1 hour, 1–2 hours, 2–4 hours, 4–8 hours, >8 hours) while for BMI and blood pressure the similar procedure was made for cohort and sex only. A composite metabolic score, defined as the standardized sum of exposures in z-scores, was created to assess combined effects of the exposures. In the models with z-scores, mid blood pressure [12] was calculated as (systolic+ diastolic blood pressure)/2 in order to avoid co-linearity. As the distribution of glucose and triglycerides was skewed, natural logarithm was applied before z-score transformation.

Cox models using exposures in z-scores were stratified by cohort and adjusted for categories of birth date, age at measurement and smoking status, model 1. In a second approach we started from model 1 using BMI and smoking and added the separate exposures, one by one, to the model in order to investigate which of the exposures attenuated each other. Finally we analysed a mutually adjusted model containing all the separate exposures, model 2. We also calculated the risk of death from RCC by a Cox model mutually adjusted for the separate z-scores, categories of birth date, age at measurement and smoking status and stratified by cohort.

To check for linear associations between increasing levels of exposure and risk, we plotted restricted cubic polynomial splines with knots at the 5^th^, 35^th^, 65^th^ and 95^th^ percentiles for exposures transformed to z-scores, using model 2 as described above. The fit of the spline model was tested versus a fit using a linear model with likelihood-ratio test. P-values <0.05 were interpreted as the cubic spline model described the risk distribution better than the linear model.

We used Wald test to test for multiplicative interaction between metabolic factors and between these factors and smoking on risk of RCC by use of z-scores. In total, 15 tests of multiplicative interactions were performed for each sex and we adjusted the significance level for multiple testing using the Bonferroni correction [13]. To investigate biological interaction between metabolic factors on risk of RCC, we calculated relative excess risk due to interaction (RERI), attributable proportion due to interaction (AP), synergy index (S) and their 95% confidence intervals using methods proposed by Andersson *et al* [14] for dichotomous variables using a cut-off at z-score = 1. In proportional hazard models like this, RERI has been suggested to be the best choice for biological interaction test [15].

Absolute risks, individualized probabilities of developing RCC for quintiles of the composite score were calculated as described by Gail *et al* [16], taking into account survival from competing risks.

### Correction for Random Error

We corrected HRs for random error, i.e. measurement error and within-person variability, by use of methods based on regression dilution ratio, similar to those described by Wood *et al* [17]. In these calculations data from subjects who had undergone repeated measurements in Me-Can were used, in total 133,820 subjects with 406,364 health examinations.

We used two methods for correction; direct adjustment of the estimated parameter using the estimated regression dilution ratio (RDR) and regression calibration. RDR was estimated as the regression coefficient in the regression models with the repeated measurement as dependent variable and the baseline health measurement as independent variable. Age at baseline, fasting time, smoking status, sex, birth year, BMI, time from date of baseline were included as fixed effects in the model and cohort was included as random effect. We used RDR in analysis of quintiles and for z-scores using model 1. In our data set, RDR was for BMI 0.90, for systolic blood pressure 0.53, for diastolic blood pressure 0.51, for glucose (log) 0.28, for cholesterol 0.66, and for triglycerides (log) 0.51. Thus, measurements of BMI had a much smaller random error than the other exposures in accordance with previous observations [18]–[20]. The correction was applied by dividing the regression coefficient computed by the Cox model with RDR for the exposure, HR_corrected_ = e^log(HRoriginal)/RDR^. In the multivariable approach when adjusting for other metabolic factors in different combinations and in model 2, we replaced the original z-score with the expected z-score given the baseline z-score and the other covariates calculated in a similar mixed linear model [21]. RDR and regression calibration were predicted at half of the mean follow up time, i.e. 6 years after baseline examination.

All statistical tests were two-sided, and p-values lower than 0.05 was considered as statistical significant. Calculations were carried out with SAS version 9.1 (SAS Institute Inc., Cary, NC, USA), STATA version 11.2 (StataCorp LP, College Station, Texas, USA), and R version 2.7.2, used for random error calculation.

## Results

The study population consisted of 278,920 men and 281,468 women with a median age at recruitment of 42 years (inter quartile range, IQR = 10 years) (**Table 1**). At baseline, overweight (BMI ≥25 kg/m^2^) or obesity (BMI ≥30 kg/m^2^) was observed in 54% of the men and 41% of the women, hypertension (systolic blood pressure ≥140 mmHg or diastolic blood pressure ≥90 mmHg) was observed in 38% of men and in 26% of women. Approximately 60% of men and 50% of women were current or former smokers. During a median follow-up of 10 years (IQR = 7 years), 592 men and 263 women were diagnosed with RCC at a median age 62 years (IQR = 14 years) and 244 men and 84 women died from RCC. RCC incidence per 100 000 person-years was 18 among men and 9 among women in the study cohort.

**Table 1:** Characteristics of the study population in the Metabolic syndrome and Cancer project (Me-Can).

		Men N (%)	Women N (%)
**Total**		278,920 (49.8)	281,468 (50.2)
**Person-years at risk**		3,503,905	3,104,255
**Cohort**	Oslo	16,694 (6.0)	–
	NCS	25,854 (9.3)	25,001 (8.9)
	CONOR	51,708 (18.5)	57,331 (20.4)
	40-y	60,543 (21.7)	67,998 (24.2)
	VHM&PP	72,219 (25.9)	85,620 (30.4)
	VIP	29,945 (10.7)	35,212 (12.5)
	MPP	21,957 (7.9)	10,306 (3.7)
**Age at measurement (years)**	<30	26,671 (9.6)	32,688 (11.6)
	30–44	152,907 (54.8)	151,773 (53.9)
	45–59	72,528 (26.0)	64,819 (23.0)
	≥60	26,814 (9.6)	32,188 (11.4)
**Smoking status**	Never-smoker	108,020 (38.7)	140,674 (50.0)
	Ex-smoker	83,727 (30.0)	71,486 (25.4)
	Smoker	87,173 (31.3)	69,308 (24.6)
**BMI (kg/m^2^)**	<25	126,140 (45.2)	166,216 (59.1)
	25.0–29.9	122,979 (44.1)	80,696 (28.7)
	≥30	29,801 (10.7)	34,556 (12.3)
**Hypertension** *		106,185 (38.1)	73,184 (26.0)
**Follow-up (years)**	<5	37,405 (13.4)	37,466 (13.3)
	5–9	110,793 (39.7)	122,556 (43.5)
	10–20	78,214 (28.0)	92,824 (33.0)
	≥20	52,508 (18.8)	28,622 (10.2)

* systolic blood pressure ≥140 mmHg or diastolic blood pressure ≥90 mmHg.

Abbreviations: Oslo study I cohort (Oslo), Norwegian Counties Study (NCS), Cohort of Norway (CONOR), Age 40-programme (40-y), Vorarlberg Health Monitoring and Prevention Programme (VHM&PP), Västerbotten Intervention Project (VIP), Malmö Preventive Project (MPP), Body Mass Index (BMI).

In analysis of quintiles adjusted for BMI and smoking, we found an increased risk of RCC among men for highest vs. lowest quintile of BMI, (HR = 1.51, 95% CI 1.13–2.03), systolic blood pressure (HR = 3.40, 95% CI 1.91–6.06) diastolic blood pressure, (HR = 3.33, 95% CI 1.85–5.99), glucose, (HR = 3.75, 95% CI 1.46–9.68), triglycerides, (HR = 1.79, 95% CI 1.00–3.21) and the composite score, (HR = 2.68, 95% CI 1.75–4.11) (**Table 2** **)**. Among women, we found an increased risk for BMI, highest vs. lowest quintile, (HR = 2.21, 95% CI 1.32–3.70) and the composite score, (HR = 2.29, 95% CI 1.12–4.68) (**Table 3** **)**.

**Table 2:** Hazard ratios of renal cell carcinoma for increasing quintile levels of exposures among **men** in the Metabolic syndrome and cancer project (Me-Can).

Exposure	Quintile	Mean (SD)	*n* cases	HR (95% CI)*	HR (95% CI)**
**BMI**	1	21.5 (1.3)	89	1.00	1.00
**(kg/m^2^)**	2	23.8 (0.8)	108	1.09 (0.80–1.48)	1.11 (0.81–1.52)
	3	25.4 (0.8)	100	0.91 (0.66–1.25)	0.94 (0.68–1.29)
	4	27.1 (0.9)	139	1.25 (0.92–1.68)	1.28 (0.95–1.73)
	5	31.7 (3.6)	156	1.47 (1.10–1.97)	1.51 (1.13–2.03)
	***P*** **_trend_**			0.0019	0.001
**Systolic blood pressure**	1	112.2 (6.3)	59	1.00	1.00
**(mm Hg)**	2	122.7 (3.7)	81	1.78 (0.94–3.38)	1.77 (0.93–3.36)
	3	129.6 (4.2)	119	1.81 (1.00–3.29)	1.77 (0.97–3.22)
	4	138.2 (4.3)	137	2.89 (1.61–5.18)	2.76 (1.53–4.99)
	5	156.4 (13.5)	196	3.68 (2.09–6.48)	3.40 (1.91–6.06)
	***P*** **_trend_**			<.0001	<.0001
**Diastolic blood pressure**	1	66.9 (5.0)	63	1.00	1.00
**(mm Hg)**	2	73.8 (2.7)	55	1.56 (0.75–3.27)	1.57 (0.75–3.29)
	3	79.5 (2.7)	95	1.72 (0.90–3.28)	1.71 (0.89–3.28)
	4	83.2 (3.1)	147	2.09 (1.14–3.82)	2.06 (1.12–3.79)
	5	95.5 (7.4)	232	3.51 (1.98–6.21)	3.33 (1.85–5.99)
	***P*** **_trend_**			<.0001	<.0001
**Glucose**	1	4.2 (0.5)	78	1.00	1.00
**(mmol/L)**	2	4.8 (0.3)	116	3.49 (1.31–9.28)	3.34 (1.26–8.89)
	3	5.1 (0.4)	122	4.41 (1.67–11.65)	4.17 (1.58–11.02)
	4	5.5 (0.4)	129	2.90 (1.11–7.58)	2.67 (1.02–6.99)
	5	6.9 (2.0)	147	4.43 (1.73–11.36)	3.75 (1.46–9.68)
	***P*** **_trend_**			0.0098	0.03
**Cholesterol**	1	4.3 (0.5)	75	1.00	1.00
**(mmol/L)**	2	5.1 (0.3)	119	1.49 (0.96–2.31)	1.43 (0.92–2.22)
	3	5.7 (0.3)	118	1.28 (0.82–1.99)	1.20 (0.77–1.87)
	4	6.3 (0.3)	145	1.63 (1.06–2.50)	1.48 (0.96–2.28)
	5	7.4 (0.8)	135	1.30 (0.84–2.02)	1.15 (0.74–1.78)
	***P*** **_trend_**			0.37	0.83
**Triglycerides**	1	0.8 (0.2)	92	1.00	1.00
**(mmol/L)**	2	1.2 (0.2)	106	1.08 (0.59–1.98)	1.01 (0.55–1.85)
	3	1.5 (0.3)	103	0.92 (0.50–1.69)	0.82 (0.44–1.50)
	4	2.1 (0.4)	132	1.55 (0.87–2.75)	1.28 (0.71–2.31)
	5	3.7 (1.7)	159	2.38 (1.37–4.15)	1.79 (1.00–3.21)
	***P*** **_trend_**			0.0002	0.01
**Composite score**	1	−1.3 (0.4)	63	1.00	1.00
	2	−0.6 (0.1)	86	1.22 (0.76–1.96)	1.21 (0.75–1.94)
	3	−0.1 (0.1)	112	1.43 (0.91–2.24)	1.41 (0.90–2.22)
	4	0.5 (0.2)	152	1.98 (1.28–3.05)	1.94 (1.26–3.00)
	5	1.4 (0.6)	179	2.74 (1.78–4.20)	2.68 (1.75–4.11)
	***P*** **_trend_**			<.0001	<.0001

* Cox regression models are adjusted for categories of birth year, age at measurement and stratified for cohort.

** Same as above but additionally adjusted for smoking and quintiles of BMI (except for BMI and the composite score).

Regression dilution ratio was used for random error correction, could be transformed back to original data by: HR_original_ = e^log(HRcorrected)*RDR^. RDR for BMI = 0.902, Systolic blood pressure = 0.525, Diastolic blood pressure = 0.497, Glucose = 0.294, Cholesterol = 0.657, Triglycerides = 0.465, Composite score = 0.688.

**Table 3:** Hazard ratios of renal cell carcinoma for increasing quintile levels of exposures among **women** in the Metabolic syndrome and cancer project (Me-Can).

Exposure	Quintile	Mean (SD)	*n* cases	HR (95% CI)*	HR (95% CI)**
**BMI**	1	20.0 (1.2)	24	1.00	1.00
**(kg/m^2^)**	2	22.2 (0.8)	28	0.92 (0.50–1.69)	0.95 (0.52–1.74)
	3	24.1 (0.8)	61	1.77 (1.04–3.01)	1.84 (1.08–3.13)
	4	26.4 (1.0)	66	1.66 (0.98–2.81)	1.74 (1.02–2.94)
	5	31.7 (3.6)	84	2.08 (1.25–3.49)	2.21 (1.32–3.70)
	***P*** **_trend_**			0.0005	0.0002
**Systolic blood pressure**	1	104.0 (5.7)	21	1.00	1.00
**(mm Hg)**	2	114.2 (3.3)	42	1.75 (0.64–4.75)	1.65 (0.61–4.49)
	3	122.3 (2.8)	44	1.59 (0.59–4.33)	1.42 (0.52–3.86)
	4	133.0 (4.9)	56	0.96 (0.36–2.55)	0.81 (0.30–2.16)
	5	155.7 (16.1)	100	2.05 (0.80–5.27)	1.58 (0.60–4.14)
	***P*** **_trend_**			0.20	0.54
**Diastolic blood pressure**	1	61.3 (4.8)	28	1.00	1.00
**(mm Hg)**	2	70.1 (3.1)	35	0.63 (0.23–1.72)	0.60 (0.22–1.65)
	3	74.5 (3.0)	31	0.86 (0.30–2.42)	0.78 (0.27–2.21)
	4	80.5 (2.7)	74	0.91 (0.37–2.26)	0.79 (0.32–1.98)
	5	92.0 (7.7)	95	1.36 (0.56–3.28)	1.06 (0.43–2.62)
	***P*** **_trend_**			0.13	0.41
**Glucose**	1	4.1 (0.5)	43	1.00	1.00
**(mmol/L)**	2	4.6 (0.3)	36	0.55 (0.12–2.49)	0.52 (0.12–2.37)
	3	5.0 (0.3)	60	1.20 (0.32–4.58)	1.10 (0.29–4.18)
	4	5.3 (0.3)	47	0.76 (0.19–3.11)	0.66 (0.16–2.71)
	5	6.5 (1.6)	77	1.62 (0.45–5.85)	1.27 (0.35–4.62)
	***P*** **_trend_**			0.30	0.54
**Cholesterol**	1	4.2 (0.4)	25	1.00	1.00
**(mmol/L)**	2	4.9 (0.2)	31	0.96 (0.43–2.15)	0.92 (0.41–2.05)
	3	5.5 (0.3)	53	1.52 (0.73–3.16)	1.40 (0.67–2.91)
	4	6.1 (0.3)	63	1.49 (0.72–3.08)	1.33 (0.64–2.74)
	5	7.3 (0.9)	91	1.83 (0.90–3.71)	1.56 (0.77–3.17)
	***P*** **_trend_**			0.04	0.11
**Triglycerides**	1	0.6 (0.1)	32	1.00	1.00
**(mmol/L)**	2	0.9 (0.1)	47	1.07 (0.40–2.83)	0.96 (0.36–2.53)
	3	1.1 (0.1)	38	0.56 (0.20–1.56)	0.45 (0.16–1.26)
	4	1.5 (0.2)	68	1.61 (0.64–4.03)	1.15 (0.45–2.93)
	5	2.5 (1.1)	78	1.71 (0.69–4.23)	1.04 (0.41–2.66)
	***P*** **_trend_**			0.07	0.56
**Composite score**	1	−1.3 (0.3)	22	1.00	1.00
	2	−0.6 (0.1)	31	0.95 (0.43–2.10)	0.94 (0.42–2.10)
	3	−0.1 (0.1)	48	1.32 (0.63–2.78)	1.31 (0.62–2.77)
	4	0.5 (0.2)	58	1.39 (0.66–2.91)	1.38 (0.66–2.89)
	5	1.5 (0.7)	104	2.30 (1.13–4.71)	2.29 (1.12–4.68)
	***P*** **_trend_**			0.0011	0.0011

* Cox regression models are adjusted for categories of birth year, age at measurement and stratified for cohort.

** Same as above but additionally adjusted for smoking and quintiles of BMI (except for BMI and the composite score).

Regression dilution ratio was used for random error correction, could be transformed back to original data by: HR_original_ = e^log(HRcorrected)*RDR^. RDR for BMI = 0.902, Systolic blood pressure = 0.525, Diastolic blood pressure = 0.497, Glucose = 0.294, Cholesterol = 0.657, Triglycerides = 0.465, Composite score = 0.688.

**Figure 1:**
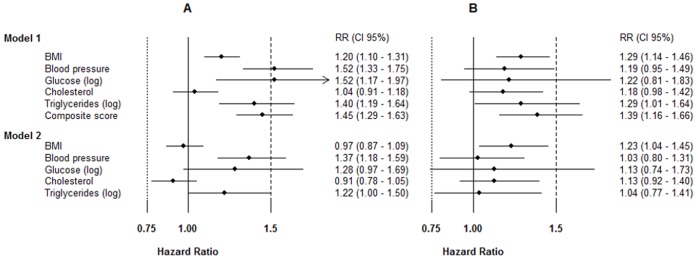
Risk of RCC by exposures in z-scores A) among men, B) among women. Model 1: Cox regression models were adjusted for smoking, categories of birth year, age at measurement and stratified for cohort. Regression dilution ratio was used for random error correction, could be transformed back to original data by: HR_original_ = e^log(HRcorrected)*RDR^. RDR for BMI = 0.902, Mid blood pressure = 0.544, Glucose (log) = 0.278, Cholesterol = 0.657, Triglycerides (log) = 0.505, Composite score = 0.688. Model 2: Cox regression models were adjusted for all single exposures, smoking, categories of birth year, age at measurement and stratified for cohort using z-scores corrected for random errors by regression calibration.

In z-score analysis among men, we found the same risk factors as found for quintile analyses using model 1 (**Figure 1a**). After including other exposures to the model the association between BMI and risk of RCC was mainly attenuated after inclusion of triglycerides but also after including blood pressure and glucose into the model, and the association between glucose and risk was attenuated by inclusion of triglycerides. Calculated per unit increase, the associations for mid blood pressure (HR = 1.37, 95% CI 1.18–1.59) and triglycerides, (HR = 1.22, 95% CI 1.00–1.50) and risk of RCC remained after adjustment for all other factors and smoking in model 2 (**Figure 1a**).

Among women, high levels of BMI and triglycerides (borderline) were associated with risk of RCC in model 1 (**Figure 1b**). When adding other metabolic factors to the model, the association between triglycerides and risk was attenuated by BMI. After adjustment for all metabolic exposures in model 2, the association between BMI were still statistically significant, (HR = 1.23, 95% CI 1.04–1.45) (**Figure 1b**).

High levels of the composite metabolic score were associated with increased risk of RCC, among men (HR per unit increase = 1.45, 95% CI 1.29–1.63) and among women (HR = 1.39, 95% CI 1.16–1.66). High levels of the composite metabolic score were also associated with risk for death from RCC, among men (HR = 1.55, 95% CI 1.29–1.85) and women (HR = 1.81, 95% CI 1.35–2.43). In analysis for death from RCC for z-scores, the only statistically significant result was the association between high blood pressure and death from RCC among men (RR = 1.33, 95% CI 1.05–1.67) (**results not shown**).

**Figure 2:**
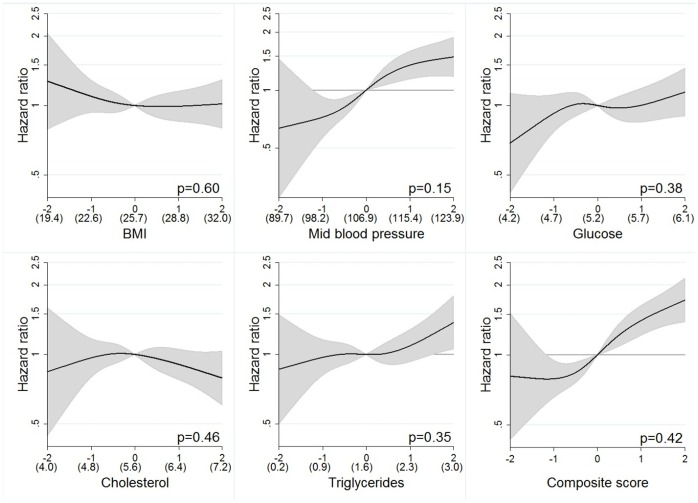
Restricted cubic splines by exposures in z-scores for men. P-values from likelihood ratio-test in the figures comparing the cubic spline polynomial with a linear model. Mean values for measured levels of exposure within parenthesis, calculated for subjects fasting >8 h for glucose, triglycerides and cholesterol. Values calibrated for random errors by regression calibration.

Trend tests over quintiles and analyses using spline models (**Figure 2** **and** **3**) indicated approximately linear associations for all metabolic factors in relation to RCC risk, which supports the use of linear models. We found no biological interaction between metabolic factors or multiplicative statistical interactions between the exposures or between exposure and smoking on risk of RCC after applying the Bonferroni correction. The absolute risks for RCC over a 20-year interval for a 40 year old man in the lowest composite score quintile was 0.18% and for a man in the highest quintile 0.34%, and corresponding risks for a man aged 60 was 0.42%, and 0.81%, respectively. For a 40 year old woman, the absolute risk of RCC were 0.10% in the lowest quintile and 0.18% in the highest quintile of the composite score, and corresponding risks for a 60 year old woman were 0.30% and 0.53%, respectively.

**Figure 3:**
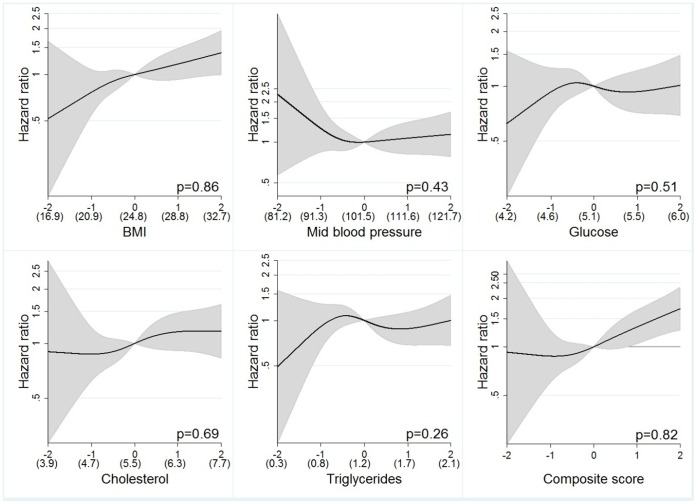
Restricted cubic splines by exposures in z-scores for women. P-values from likelihood ratio-test in the figures comparing the cubic spline polynomial with a linear model. Mean values for measured levels of exposure within parenthesis, calculated for subjects fasting >8 h for glucose, triglycerides and cholesterol. Values calibrated for random errors by regression calibration.

## Discussion

In this large prospective cohort study, we found that high levels of several metabolic factors, both separately and combined, were associated with an increased risk of RCC. Of the single factors, high levels of BMI, blood pressure, glucose and triglycerides were among men and BMI among women associated with increased risk of RCC. Several of these factors may be part of the same biological pathway, but we found that blood pressure and triglycerides among men and BMI among women independently increased the risk of RCC. No evidence was found for multiplicative statistical or biological interactions between these factors, smoking and risk of RCC and the associations between metabolic factors and risk of RCC were approximately linear.

Strengths of our study include the large number of participants in combination with high quality registers with almost complete capture of cancer diagnoses [22]–[24], and the availability of repeated measurements allowing us to correct risk estimates for random error. The main limitation of our study was a lack of data on medications that may have influenced the metabolic factors, and also the crude classification of smoking status that may have resulted in some residual confounding.

By transforming the exposures to the same scale using z-scores, we were able to compare the risk estimates on the same scale. By use of spline polynomials and tests for linearity we assessed linear association between exposure and risk, and these results support the use of continuous variables. Consequently, we mainly used the z-scores as continuous variables in the analysis and used different adjustments in the models to investigate interplay between the metabolic factors, and to assess if the exposures were independently associated with risk. We used both multiplicative models and tests for biological interaction to explore interactions between metabolic factors and between these factors and smoking.

Among men, our findings for BMI and blood pressure are in accordance with results from earlier studies [6], [25]–[27]. Similar to previous data, the results for BMI were attenuated after adjustments of other metabolic factors [26] and high blood pressure was independently associated with risk of RCC [6]. Our findings for glucose levels and risk for RCC are in line with previous reports [7], [9] when comparing the results from models adjusted for BMI. For blood lipids, previous reports showed no association for high cholesterol levels [7], [8] and an association for high levels of triglycerides [7] with risk of RCC, similar to our data.

The association between BMI and RCC was stronger in women than in men, in line with a recently published meta-analysis by Mathew *et al* [30]. In contrast to several previous cohort studies [26]–[28], [31] we did not observe an association between blood pressure and RCC among women. This cannot be explained by a low power since the number of female RCC cases was more than twice than those in the before mentioned cohort studies. High serum cholesterol was not associated with risk of RCC in women, in line with results from a large Korean study [8]. To the best of our knowledge, no previous cohort study has so far investigated the effect of blood glucose levels or triglycerides on the risk of RCC separately in women. However, previous studies have reported an increased risk of RCC in women with diabetes type 2 [5], [32].

No multiplicative statistical interaction was found in the data, and no evidence of biological interaction, which would indicate departure from additive effects. Thus, the interaction between metabolic factors on risk of RCC are on an additive scale, which is in accordance with previous studies [5], [7], [27], [33], [34],with exception of one case-control study that found multiplicative interaction between obesity and hypertension in women but not in men [34].

Smoking is an established risk factor for RCC and we found HRs around 1.4–1.5 for smokers versus never smokers among both men and women, similar to previous findings [35]. However, no effect modification due to smoking status was found. Two previous study plotted spline functions for increasing levels of BMI and systolic and diastolic blood pressure and risk of RCC. For BMI that study concluded in line with our data a steady increase of RCC with increasing levels of BMI [29] the other study reported, in contrast to our data, a positive but non-linear dose-response between blood pressure and RCC [28].

Our results indicate that high BMI, blood pressure, glucose and triglycerides among men and BMI among women are associated with RCC. Several of these factors may interplay on biological pathways, but we found that blood pressure and triglycerides among men and BMI among women were independent risk factors. Possible biological mechanisms that may link metabolic abbreviations to risk of RCC are insulin-like growth factor (IGF-1) [36], lipid peroxidation [37] and metabolic changes within the renal tubule [38].

From a public health perspective our data add some further motivation to control metabolic factors in addition to the decreased risk of cardiovascular disease and diabetes type 2. However, the absolute increase in risk of RCC for high levels of a composite score of all five metabolic factors was modest.

In conclusion, high levels of BMI, blood pressure, glucose, triglycerides among men and BMI among women is associated with increased risk of RCC, but no interaction was found between these factors and RCC.
