# Correction: Metabolic Factors Associated with Risk of Renal Cell Carcinoma

**DOI:** 10.1371/annotation/bb4481d0-a1ac-4fd9-aa57-e267f719a189

**Published:** 2013-04-22

**Authors:** Christel Häggström, Kilian Rapp, Tanja Stocks, Jonas Manjer, Tone Bjørge, Hanno Ulmer, Anders Engeland, Martin Almqvist, Hans Concin, Randi Selmer, Börje Ljungberg, Steinar Tretli, Gabriele Nagel, Göran Hallmans, Håkan Jonsson, Pär Stattin

There was an error in the funding statement. The correct funding information is:

This study was supported by grants from Lion's Cancer Research Foundation (http://www.cancerforskningsfond-umea.lions.se/), Umeå University, Sweden (LP 09-1799). Funding for the Me-Can project was obtained from the World Cancer Research Fund (WCRF UK) (http://www.wcrf-uk.org/) for grant 2007/09 and from Wereld Kanker Onderzoek Fonds (WCRF NL) (http://www.wcrf.nl/) for grant R2010/247, as part of the WCRF International grant programme, and the Swedish Cancer Foundation (http://www.cancerfonden.se/sv/Information-in-English/)(2010/628). The funders had no role in study design, data collection and analysis, decision to publish, or preparation of the manuscript.

